# Radioimmunotherapy Combined With Low-Intensity Ultrasound and Microbubbles: A Potential Novel Strategy for Treatment of Solid Tumors

**DOI:** 10.3389/fonc.2021.750741

**Published:** 2021-10-22

**Authors:** Binwei Lin, Huan Du, Jinjia Fan, Dan Huang, Feng Gao, Jie Li, Yu Zhang, Gang Feng, Tangzhi Dai, Xiaobo Du

**Affiliations:** ^1^ Department of Oncology, Nuclear Medicine Laboratory of Mianyang Central Hospital, Mianyang Central Hospital, Mianyang, China; ^2^ Department of Oncology, Affiliated Hospital of North Sichuan Medical College, Nanchong, China; ^3^ Radiology Department, Mianyang Central Hospital, Mianyang, China

**Keywords:** radioimmunotherapy, ultrasound, microbubble, drug delivery, blood-tumor barrier

## Abstract

The prognosis of advanced malignant tumors is very poor, and effective treatment is limited. Radioimmunotherapy (RIT) is a novel treatment method. However, its anti-tumor effect is relatively low in solid tumors, which is mainly due to the blood-tumor barrier preventing RIT from penetrating the tumor, resulting in an insufficient dose. Low-intensity ultrasound with microbubbles (USMB) has proven capable of opening the blood-tumor barrier. The combination of the two technologies may overcome the poor anti-tumor effect of RIT and promote the clinical application of RIT in solid tumors. In this article, we reviewed the current research status of RIT in the treatment of solid tumors and the opportunities and challenges of USMB combined with RIT.

## Introduction

Cancer is the leading cause of human death (1). Currently, surgery, radiotherapy, chemotherapy, targeted treatment, immunotherapy, and other therapeutic modalities are the main treatment methods for patients with cancer. Although comprehensive treatment can effectively control cancer and prolong patients’ survival, approximately 70% of patients still have disease recurrence and progression (2). Therefore, new treatments are urgently needed. Radioimmunotherapy (RIT) is a promising novel anti-tumor therapy that utilizes the combination of monoclonal antibodies with radionuclides. Monoclonal antibodies combined with tumor-specific antigens can kill tumor cells through antibody-dependent cell-mediated cytotoxicity (3). Moreover, radionuclides can self-decay, inducing ionizing radiation, which in turn potentiates the anti-tumor effect. The advantage of RIT is that it retains the targeting and inherent anti-tumor effect of monoclonal antibodies. In addition, monoclonal antibodies transport radionuclides to the tumor site, which allows the ionizing radiation to target and kill the tumor cells. Presently, α-rays (helium nuclear rays) and β-rays (electron rays) produced by nuclide decay are the main rays used in anti-tumor therapy (4). As high linear energy transfer (LET) particles, α-rays transfer higher energy along the particle track, have a higher probability of causing irreparable DNA damage than β-rays (low LET particles) (5). Moreover, the radionuclides used for antibody labeling are characterized by the ability to emit short-range rays (β and α rays), such as 177Lu, 131I, 223Ra, and the maximum radiation radius of the rays ranges from 50 μm to 2.3 mm (6). Therefore, the normal tissues around the cancer are left almost unirradiated, making the toxicities related to radionuclide radiation extremely low. RITs, such as 131I tositumomab (7), 131I rituximab (8), and 90Y teimozumab (9) have been approved by the United States Food and Drug Administration for the treatment of hematological tumors. However, the application of RIT in solid tumor therapy remains a challenge. Solid tumors, which are different from hematological tumors, have blood-tumor barriers (BTBs). BTBs are composed of proliferating tumor cells, tumor stroma, and angiogenic vessels (10), and BTB could limit the penetration of RIT into the tumor (11, 12). Besides, solid tumors are more insensitive to radiation compared to hematological tumors (13). Even when RIT reaches the maximum tolerated dose (MTD), the RIT dose in the tumor is insufficient. As a result, the RIT cannot effectively kill the solid tumor cells.

Low-intensity ultrasound with microbubbles (USMB) is a drug delivery technology often used in anti-tumor treatment with cytotoxic and monoclonal antibody drugs. Low-intensity ultrasound sonicates the local tumor, acts on the endogenous and exogenous microbubbles in tumor blood vessels, produces a cavitation effect, and increases the permeability of solid tumor tissue at the sonication site (10, 14). Some preclinical studies have confirmed that USMB can increase the concentration of anti-tumor drugs in the sonication tumor area, and does not affect normal tissues without sonication; thus, improving the therapeutic ratio of anti-tumor drugs (15–21). Recently, Marie et al. (22) reported that USMB can promote the uptake of antibodies by the tumor tissue in subcutaneous breast cancer models in mice, and speculated that antibody-based therapies combined with USMB may be therapeutically beneficial. Studies have also shown that ultrasound can enhance the radiosensitivity of tumor cells (23, 24). Therefore, USMB combined with RIT may overcome the limitations of RIT alone in the treatment of solid tumors and promote the clinical transformation of RIT in the management of solid tumors. RIT coupled with high LET radionuclides have a stronger anti-tumor effect, which is discussed in detail in another review (4). This review mainly discusses the feasibility of USMB combined with RIT, the existing problems, and a novel combination strategy to improve the anti-tumor effect of RIT in the treatment of solid tumors, and provides a theoretical basis and new ideas for follow-up studies.

## Clinical Research Status of RIT in Solid Tumors

The concentration of RIT in solid tumors compared to that in blood tumors is insufficient due to the BTB. Moreover, solid tumors are relatively insensitive to radiation and require higher drug doses to effectively kill tumors. As a result, tumor regression is not apparent even if RIT reaches the MTD.

We searched PubMed, Cochrane Central Register of Controlled Trials (CENTRAL), ClinicalTrials.gov, and Embase databases for clinical studies on RIT that were published in the past 15 years, and found that 39 such studies used RIT in the treatment of solid tumors. The main cancer types treated were colorectal cancer, pancreatic cancer, breast cancer, gastric cancer, and liver cancer. However, these clinical studies are mainly phase I/II studies, with 19 phase I studies and 19 phase II studies. There was only one phase III study, which involved patients with postoperative ovarian cancer and which utilized an intraperitoneal administration of RIT (25). In this phase III study, a total of 447 patients were randomly divided into an experimental group (intraperitoneal injection of RIT combined with standard adjuvant treatment group) and a control group (standard adjuvant treatment group). The result showed that the recurrence rate of the experimental group was significantly lower than that of the control group. However, it should be noted that in this phase III study, since the tumor was removed as much as possible, and the route of administration of RIT is intraperitoneal perfusion, there was no BTB in the route of transportation of RIT (25).

In the presence of the BTB, the anti-tumor effect of RIT is insufficient. Street et al. reported the results of a phase I clinical study on RIT in patients with advanced colorectal cancer. The selected target was the carcinoembryonic antigen, and the RIT used was 131I-chTNT-1/B. The MTD was 58.09 MBq/kg, and the main dose-limiting toxicity (DLT) was myelosuppression. When the MTD was reached, the objective remission rate (ORR) was 0% (26). Giraudet et al. reported a phase I study on the anti-tumor effect of 90Y-OTSA-101 on patients with advanced sarcoma. A total of eight patients were included, and the target was FZD10. The study found that hematotoxicity was the main treatment-related toxicity of grade 3 and higher grades. The follow-up results showed that no patient achieved objective remission. The authors believe that even if an additional phase II study is needed, radionuclides need to be replaced to overcome the problem of treatment insensitivity (27). Stillbroer et al. enrolled 23 patients with advanced renal cell carcinoma and used 177Lu-girentuximab to target carbonic anhydrase IX. The MTD was 2,405 MBq/m^2^, and the main DLT was myelosuppression. Unfortunately, the therapeutic effect was still unsatisfactory, and only one patient achieved partial remission (PR) (28). Regarding the patients with BTB, the anti-tumor effect was poor (**Table 1**). The main reason may be that the RIT dose that reaches the tumor is insufficient. Even when the dose reaches the MTD, RIT cannot kill the tumor tissue effectively.

**Table 1:** Summary of the clinical research on RIT in the treatment of solid tumors in the presence of the BTB.

Author	Disease	Number of participants	Study phase	RIT	Primary endpoint	Secondary endpoint(s)
Street et al. (26)	colorectal cancer	21	I	131I-chTNT-1/B	MTD:58.09 MBq/kg	CR:0%, PR:0%
Giraudet et al. (27)	synovial sarcoma	8	I	90Y-OTSA-101	haematological disorders were most common Grade ≥ 3 AEs	objective response:0%; SD: 3/8(37.5%)
Stillebroeret al. (28)	renalcell carcinoma	23	I	177Lu-girentuximab	MTD:2405 MBq/m(2)	PR:1/23 (4.3%), SD:17 (73.9%)
Picozzi et al. (29)	pancreatic carcinoma	29 vs 29	Ib	90Y-clivatuzumab+ gemcitabine vs 90Y-clivatuzuma	Cytopaenias were the significant toxicities.	PR: 2/29 (6.9%) VS 0/29 (0%), mOS:7.9 months vs 3.4 months
Meyer et al. (30)	Gastrointestinal carcinomas	12	I	131I-A5B7	dose-limiting AE:myelosuppression	PR:0%, SD:3/10 (30%)
Gulec et al. (31)	pancreatic carcinoma	20	I	90Y-hPAM4	MTD:20 mCi/m(2)	PR:3/20 (15%)
Sultana et al. (32)	pancreatic carcinoma	25	I/II	131I-KAb201	MTD:50 mCi	Overall response rate: 1/18 (6%), mOS:5.2 months
Muselaers et al. (33)	renal Cell Carcinoma	14	II	177Lu-girentuximab	SD: 57%, PR: 7%	grade 3-4 myelotoxicity in most patients
Tagawa et al. (34)	prostate cancer	49	I/II	177Lu-J591	recommended phase 2 doseswere 40 mCi/m2 and 45 mCi/m2 ×2	PR:0, SD:14/22 (60.8%)
						mOS: 42.3 months
Molina et al. (35)	prostate cancer	6	I	177Lu-J591	grade 4 neutropenia:2(3%), grade 4 thrombocytopenia:3(50%)	177 Lu-J591 targeted all tumor sites

BTB, blood tumor barrier; CR, complete response; PR, partial response; SD, stable disease; mOS, median overall survival; MTD, maximum tolerance dose; AE, adverse event.

To improve the anti-tumor effect of RIT in solid tumors, the current strategies are as follows: (1) select RIT that can radiate α-rays; (2) RIT combined with a radiosensitizer, such as gemcitabine; (3) intratumoral injection of RIT; and (4) intra-arterial injection of RIT.

Allen BJ et al. reported the results of a phase I study on (213)Bi-cDTPA-9.2.27 in the treatment of 38 patients with advanced melanoma. The dose range of RIT was 46–925 MBq, and the MTD was not reached. Of the enrolled patients, 10% achieved PR, 40% had a stable disease condition, and the median overall survival (OS) time was 8.9 months (36). (213)Bi can emit α-rays, which have a strong tumor-killing ability and short radiation radius. (213)Bi can kill tumor cells more effectively when compared with low LET rays. Simultaneously, normal tissues outside the radiation radius can be protected from radiation. However, further phase II study was not conducted. The main reason may be that the production of nuclides emitting α-rays is difficult and expensive. In addition, because the radiation radius of α-rays is only 50–100 nm (6), to kill tumor cells, RIT needs to penetrate the tumor cells. This requires RIT to be internalized by tumor cells after binding with tumor surface antigens, rather than be lysosomally cleaved inside the tumor; that is, it requires that the connection between the nucleon and antibody is extremely stable. The complex production process and high stability requirements of this kind of RIT may be the key factors limiting its clinical application. Picozzi et al. reported a study comparing RIT combined with gemcitabine and RIT alone. A total of 58 patients with pancreatic cancer who failed second-line treatment were included and assigned to two groups with a ratio of 1:1. The results showed that two patients in the combined treatment group and none of the patients in the control group achieved PR. The median OS in the combined group was significantly longer than that in the RIT alone group (7.9 months *vs*. 3.4 months). However, the incidence of hematotoxicity among the ≥ grade 3 patients in the combined treatment group was significantly higher than that in the RIT group alone (29). The intratumoral injection of RIT is an effective way to avoid the hindered penetration into the tumor due to the BTB. Although the intratumoral injection of RIT can improve the therapeutic effect on the tumor (37), this is an invasive treatment strategy and may cause tumor spread. Chen et al. reported a phase I/II clinical study that included 134 patients with hepatocellular carcinoma who were treated with hepatic arterial infusion of RIT to evaluate the safety and effectiveness of RIT. The results showed that the maximum safe dose of RIT was 27.75 MBq/kg, six patients (8.22%) achieved local remission, and that the 21-month survival rate was 44.54% (38). The advantage of the intra-arterial injection of RIT is that the drug is targeted into the tumor, which avoids the process of re-aggregation after systemic circulation and reduces the concentration of RIT in non-tumor tissues. Therefore, the concentration ratio of RIT in the tumor and blood increases. However, this treatment can only be applied to patients with a limited number of metastases and definite blood supply arteries.

The distribution of RIT in tumor and normal tissues is mainly determined by antibodies. Understanding the metabolic process of antibodies *in vivo* is very important to improve the concentration ratio of RIT in tumor and normal tissues. The distribution of antibodies in tumor tissue is related to the presence of the BTB, antigen concentration, binding affinity, antigen internalization, and systemic clearance. The BTB is an important factor that affects the entry of RIT into the tumor, including tumoral vascular endothelial cells and the extracellular matrix. Antigen concentration is another factor affecting the distribution of RIT in the tumor. Ideally, the optimal antigen for RIT should be highly expressed in tumor cells (usually >100,000 sites per cell) and is not expressed in normal tissues (4). The affinity of antigens for RIT and the internalization ability of the antigen-antibody complex also needs to be considered. The strong binding ability of antigens to RIT and the rapid internalization after binding are not conducive for RIT to reach tumor cells far away from blood vessels (39). Finally, RIT with a high systemic clearance rate is not conducive to the uptake of RIT by tumor cells (40). Generally, antibodies with small molecular weights are more capable to break through the BTB. However, due to faster systemic clearance, the actual effective uptake by the tumor is reduced, which is not conducive to anti-tumor treatment (4). In addition, tumor-stromal pressure is also an important factor that prevents RIT from penetrating the tumor. Tumor growth needs an abundant blood supply, but because the endolymphatic drainage is relatively insufficient, there is high stromal pressure, which limits the penetration of RIT into the tumor (10).

## USMB Can Penetrate the BTB and Increase the Concentration of RIT in Solid Tumors

As a noninvasive, convenient, and economical method, USMB can open the BTB by sonicating the tumor (12). Studies have confirmed that it can promote the deposition of anti-tumor-targeted drugs in tumors, improve the drug concentration in local tumors, and enhance the anti-tumor effect (22, 41).

The first barrier to prevent RIT from penetrating the tumor is blood vessels. When ultrasound acts on the blood, it can produce a cavitation effect, which is required to reach the cavitation threshold. Research shows that the cavitation threshold is inversely proportional to the square root of ultrasound frequency, and low-frequency ultrasound is more likely to induce a cavitation effect (42). Ultrasound can produce air bubbles by changing the internal pressure of blood. There are two forms of air bubbles under the mechanical action of ultrasound. One is stable cavitation, wherein the size of air bubbles changes with the change in pressure, forming local traction on the vascular wall. The other is inertial cavitation, wherein bubbles rupture, which produces shock waves around the bubbles that damage the blood vessel wall (43). These two forms of cavitation affect the connection of vascular endothelial cells, increasing the gap between these cells and the reversible or irreversible damage of some endothelial cell membranes, providing an effective channel for RIT to cross blood vessels. Microbubbles are a kind of air particle with a diameter of 1–4 μm. Due to the presence of microbubbles in the blood, the cavitation threshold is reduced (12). Therefore, USMB can enhance the cavitation effect based on ultrasound and further promote the crossing of tumor vessels by RIT. Similarly, after RIT crosses the blood vessels, it also needs to pass through the interstitial tissue (such as proteoglycans and collagen) between tumor cells and blood vessels and infiltrate inflammatory cells and other structures to produce local pressure changes *via* the cavitation effect, to increase the internal permeability of the tumor and promote the uptake of RIT by the tumor (44).

Recently, Marie et al. studied the effect of USMB on the tumor uptake of monoclonal antibodies using bilateral subcutaneous implanted tumor mice models. Antibody and microbubbles were injected from the tail vein before ultrasound sonication, and the only unilateral tumor was sonicated. They found that the concentration of antibodies on the ultrasound sonication side was significantly higher than that on the contralateral tumor without ultrasound sonication (22). Brighi et al. used focused ultrasound guided by magnetic resonance imaging to open the blood-brain barrier (BBB) and promote the uptake of RIT in tumor tissue of glioma models *in situ* and found that the concentration of RIT in tumor tissue at the ultrasound sonication site was significantly increased (45). Tran et al. also explored whether USMB can enhance the permeability of the BBB and promote the penetration ability of RIT through the BBB. The results showed that RIT in the ultrasound sonication area of the mouse brain was significantly increased (46).

## Other Potential Advantages of USMB Combined With RIT: Increased Radiosensitivity

Since the important mechanism of RIT in killing tumor cells is to use the radiation produced by decay to kill the tumors, the radiosensitivity of tumor tissue is one of the key factors that affect the anti-tumor effect. Some preclinical studies have confirmed that USMB can improve the radiosensitivity of prostate cancer (47), bladder cancer (48), nasopharyngeal carcinoma (49), fibrosarcoma (50), and breast cancer (51). This means that only a low RIT dose is needed to achieve the ideal anti-tumor effect after USMB treatment. Since the apoptosis of endothelial cells is related to tumor radiosensitivity (52), the apoptosis of vascular endothelial cells and interruption of tumor blood vessels induced by USMB are considered to be the possible mechanisms for enhancing tumor radiosensitivity (53). The up-regulation of ceramide expression induced by USMB enhances the radiosensitivity of prostate tumors after USMB treatment, which may make it an important factor in inducing the apoptosis of vascular endothelial cells (54, 55). In addition, the up-regulation of angiotensin II and its receptor, AT1R, after USMB treatment may be related to the increase of radiosensitivity (49).

## Problems to Be Solved Before Combining RIT and USMB

Although studies have confirmed that USMB can enhance the permeability of tumor tissue and increase the concentration of RIT in the tumor (45, 46), there are still many problems that need to be solved.

### Ultrasonic Parameters

Marie et al. explored the effect of ultrasound with different pulse lengths on RIT uptake and found that the promotion effect of long pulse ultrasound with 5000 cycles was the weakest. At 4 h after sonication, the promotion effect of ultrasound on tumor uptake of RIT disappeared, while the promotion effect of medium pulse ultrasound with 125 × 40 cycles and short pulse ultrasound with 500 × 10 cycles disappeared until 24 h (22). Qin J et al. summarized the ultrasonic parameters and recommended ranges affecting drug delivery efficiency, including ultrasonic frequency (0.4–3 MHz), ultrasonic intensity (0.3–3 W/cm^2^), mechanical index (0.2–1.9), duty cycle (<1–90%), and sonication time (10 s–30 min). These data were derived from the ultrasonic parameters used in published experiments (10).

The optimal ultrasound parameters may maximize the efficiency of drug delivery. Sorace et al. explored the optimal ultrasound parameters and the effect of different ultrasound parameters on the uptake of paclitaxel by breast cancer cells (56). The default ultrasonic parameters were as follows: frequency of 1 MHz, sonication time of 300 s, mechanical index of 0.5, pulse repetition period of 0.01 s, duty cycle of 20%, microbubble concentration of 14 million MBS/mL, and total amount of 50 µL. Other parameters were changed under the default conditions, and a single variable was used for the experiment. Different frequencies (0.5, 1.0, and 2.25 MHz), sonication times (15, 60, 300, and 600 s), mechanical indices (0.1, 0.5, 1.0, and 2.0), pulse repetition periods (0.01, 0.1, and 1.0 s), and microbubble quantities (10, 50, and 250 µL) were selected. The results showed that optimal ultrasound frequency is 1 MHz, mechanical index is 1, pulse repetition period is 0.01 s, and sonication time is 5 min. At the same time, with the increase of microbubbles, the drug uptake of breast cancer cells also increased (56). However, the aforementioned parameters may only provide a reference for USMB to open cell membrane permeability, and the optimal ultrasound parameters for opening BTB need further study. Sorace et al. found in subsequent animal experiments that 2 min after injection of paclitaxel and microbubbles into breast cancer animal models, different mechanical indices (0.1, 0.5, 1.0, and 2.0) had a significant impact on the treatment effect, and the mechanical index of 0.5 had the best anti-tumor effect (56). Liu et al. explored the effects of different ultrasound intensities on the transmission of 1,3-bis (2-chloroethyl) - 1-nitrosourea. Glioma *in situ* mice models were used. The ultrasound parameters were a frequency of 400 kHz, pulse length of 10 ms, pulse frequency of 1 Hz, and sonication time of 30 s. Different ultrasound intensities (0.45, 0.62, 0.98, and 1.35 MPa) were selected. The results showed that 0.62 MPa was the most effective in opening the BBB (57).

However, the drugs delivered by these experiments were mainly chemotherapeutic drugs and genes (10) which have different physical and chemical properties from RIT. Since the biodistribution of RIT is mainly determined by antibodies, **Table 2** summarizes the relevant parameters of USMB that promote tumor antibody uptake, which may be more useful for USMB combined with RIT. Future experiments are required to determine the optimal ultrasound parameters to ensure that RIT used in conjunction with USMB can penetrate tumor tissue more effectively under specific ultrasound parameters.

**Table 2:** Summary of ultrasound parameters related to USMB promoting antibody delivery.

Author	Model	Antibody	Ultrasonic parameters						Effect
			Frequency	Intensity	Mechanical index	Pulse parameters	Duty cycle	Sonication time	
Marie et al. (22)	subcutaneous/mice	anti-CD73 mAb	1 MHz	850 kPa	unknown	5000 cycle pulses given every 5 s	unknown	5min	enhanced
Caterina et al. (45)	glioma *in situ*/mice	EphA2-4B3 antibody	1.1 MHz	0.85 Mpa	unknown	10 ms focused ultrasonic bursts	unknown	2min	enhanced
Centelleset al. (58)	subcutaneous/mice	Trastuzumab	1.0 MHz	unknown	unknown	unknown	99.90%	3-5min	enhanced
Park et al. (59)	Brain metastasis *in situ*/mice	Trastuzumab	690 kHz	0.69 MPa	unknown	unknown	unknown	–	enhanced
Heath et al. (60)	subcutaneous/mice	Cetuximab	1.0 MHz	unknown	0.5	pulse repetition period: 5s	20%	5min	enhanced
Vu et al. (46)	Normal brain/mice	Cetuximab	1.5 MHz	520 kPa	unknown	unknown	69%	127 s	enhanced
Goutal et al. (61)	Normal brain/mice	erlotinib	1.5 MHz	0.6 MPa	unknown	continuous waves	unknown	5 min	not enhanced

### Selection of Antibodies

Some types of antibodies may not be effectively delivered by USMB. Goutal et al. found that while USMB can open the BBB, it cannot enhance the uptake of erlotinib in brain tissue. The authors inferred that this may be due to erlotinib utilizing the active transport mode and ABC transporters playing a key role in the transport process. When ABC transporters were fully inhibited, the concentration of 11C-labeled erlotinib in brain tissue increased significantly (61). Although USMB opened the BBB, erlotinib did not penetrate the brain tissue through these new channels. Therefore, when USMB is used to promote the uptake of RIT by tumor tissue, the type of antibodies is very important. However, for the selection of antibodies, the focus of attention is on whether they can specifically bind to tumor-specific antigens. Active research on the transfer pathway of antibodies *in vivo* may be the key to realizing the optimal parameters in the combination of USMB and RIT.

### Others

Tumor heterogeneity may be another important factor, which may lead to an uneven uptake of RIT by the tumor after ultrasound sonication. Brighi et al. found that the increase of RIT uptake after ultrasound mainly appeared in the T1-enhanced area, meaning that the uptake of RIT could be enhanced by ultrasound sonication only when the BBB was damaged (45). Low-dose RIT areas may become the center of tumor recurrence. The flow of antibodies across blood vessels is mainly through convective transport, and the level of blood perfusion in tumors is an important factor that affects convective transport (62). Ultrasound-enhanced antibody uptake was mainly in the high perfusion area (62). The size of tumor blood vessels is also an important factor that affects the flow of antibodies across blood vessels. With the increase in the diameter of blood vessels, the flow ability of antibodies across blood vessels increased and reached saturation when the diameter of blood vessels exceeds 200 nm (62). Improving the uptake of RIT in tumor areas insensitive to ultrasound to reduce the recurrence rate after RIT treatment is another challenge we need to pay close attention to.

## How to Implement USMB

**Figure 1:**
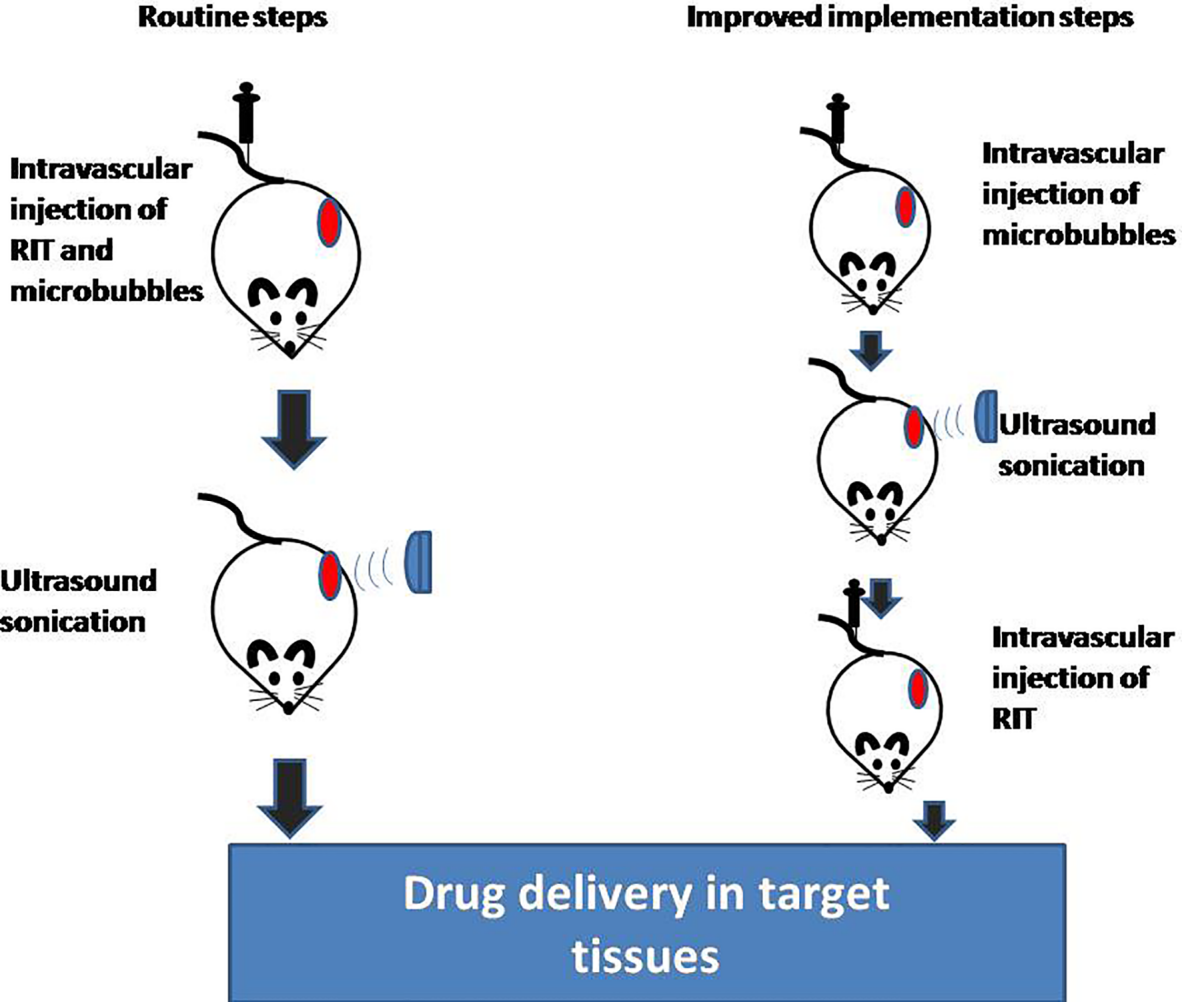
Implementation steps of USMB and RIT. USMB, ultrasound with microbubbles; RIT, radioimmunotherapy.

The routine steps used in preclinical studies of USMB to promote drug delivery are as follows: 1. intravascular injection of drugs and microbubbles; 2. ultrasound sonication; and 3. ultrasound-induced sonoporation and localized drug uptake (**Figure 1**). It should be noted that the drugs delivered in the aforementioned steps are chemical drugs [paclitaxel (56, 63) and doxorubicin (64, 65)] and genes [such as DNA (66, 67) and RNA (68)]. Compared to these drugs, RIT consists of antibodies, linkers, and radionuclides. The combined stability of RIT may be destroyed by USMB, and radionuclides may not be targeted to the tumor site, resulting in the decrease of the anti-tumor effect and the increase of the incidence of toxicity. To enhance the drug delivery of RIT using the conventional method, it needs to be confirmed that the stability of RIT will not be damaged by USMB. Since it is considered that ultrasound can reversibly open the BTB, and current studies show that the reversible BTB opening time is at least 24 h (22, 45, 69), a feasible implementation process of USMB is as follows: 1. intravenous injection of microbubbles; 2. ultrasound sonication; 3. intravascular injection of RIT; and 4. opening the BTB and promoting RIT targeting into tumor tissue (**Figure 1**). Injection of RIT immediately after ultrasound can prevent the effect of USMB on RIT. Simultaneously, since the BTB is left open after USMB, it does not affect the drug deposition of RIT. However, some problems also need to be solved before this improved implementation can be utilized. First, it is unclear whether USMB affects tumor cell antigens and subsequently affects antigen-antibody binding. Second, studies have shown that USMB can change Ca^2+^ distribution (70) and temperature (71) in the tumor microenvironment, which may affect the affinity of antigens and antibodies. Finally, after RIT binds to cell membrane antigens, part of RIT is internalized by cells before the antibodies are degraded in lysosomes. The radionuclides carried by RIT will convert to a free state and may flow out of tumor cells through exocytosis (72). The ideal situation is that radionuclides in a free state will not be released by tumor cells. Radionuclides can continuously target and kill tumor cells. At the same time, radionuclides can be restricted in tumor cells to avoid normal tissue damage. However, the effects of USMB on the internalization of RIT and the re-efflux of radionuclides remain unclear.

## Conclusion

In summary, USMB combined with RIT may be a promising combination strategy. Opening the BTB through USMB can effectively solve the problem of insufficient RIT dose in tumors and improve the prognosis of patients. However, there are still some problems that need to be solved before this technology can be clinically applied, such as the selection of ultrasound parameters and antibodies and the exploration of tumor heterogeneity. These problems will become prominent topics of research in the future.

## Author Contributions

BL and HD draft the manuscript. JF, DH, FG, JL, YZ, GF, and TD participated in the data review and collection for the study. XD conceived of the study, and participated in its design and coordination. All authors contributed to the article and approved the submitted version.

## Conflict of Interest

The authors declare that the research was conducted in the absence of any commercial or financial relationships that could be construed as a potential conflict of interest.

## Publisher’s Note

All claims expressed in this article are solely those of the authors and do not necessarily represent those of their affiliated organizations, or those of the publisher, the editors and the reviewers. Any product that may be evaluated in this article, or claim that may be made by its manufacturer, is not guaranteed or endorsed by the publisher.
